# Experiences of healthcare staff in forensic care facilities supporting sexual violence survivors, in Tshwane, South Africa

**DOI:** 10.4102/curationis.v46i1.2374

**Published:** 2023-05-26

**Authors:** Moreoagae B. Randa, Julie McGarry

**Affiliations:** 1 Department Nursing Science, Faculty of Health Care Science, Sefako Makgatho Health Sciences University, Pretoria, South Africa; 2 Health Sciences School, University of Sheffield and Sheffield Teaching Hospitals NHS Foundation Trust, Sheffield, United Kingdom

**Keywords:** forensic care, forensic care centre, healthcare staff, sexual violence, victims, survivors

## Abstract

**Background:**

Sexual violence is a persisting global epidemic that is constantly increasing on a large scale. The rate of sexual violence in South Africa is one of the highest in the world; and it has been reported to appear socially normalised and acceptable.

**Objectives:**

The study aimed to explore and describe the experiences of healthcare staff working in forensic care centres (FCCs) in Tshwane, South Africa.

**Method:**

A qualitative approach was followed incorporating focus group interviews with a range of healthcare staff based in the two FCCs. Non-probability purposive sampling was done. Data analysis was informed by the Analytic Hierarchy Model which comprised of three steps: data management, descriptive accounts and developing explanatory accounts.

**Results:**

Three main themes emerged as, (1) help them to do away with the idea of self-blame: everyday work; (2) barriers to the accessibility of care: seeking alternative traditional remedies *(muti)* from traditional healers and working in an unconducive environment and (3) compassionately sick at times: Emotional impact of forensic care work.

**Conclusion:**

The findings revealed that the healthcare staff are often working in difficult circumstances and that both professional and societal factors mediate against the provision of care and support for survivors. Greater attention is needed both in terms of service development and wider challenges to pervading societal norms surrounding violence against women.

**Contribution:**

The study highlighted the need for training, improved management support and debriefing sessions.

## Introduction

Gender-based violence that includes actual or threatened physical, sexual or psychological harm, coercion or deprivation of liberty is a significant global public health and societal problem and a universal human rights issue (World Health Organization [WHO] 2021; United Nation 1993). Global estimates indicate that one in three women will experience physical or sexual violence in their lifetime (WHO 2022). Gender-based violence exerts a detrimental impact on the lives and health of all those who experience abuse and this includes children (Westmarland & Kelly 2013). In recognition of the global impact of violence against women, the WHO Sustainable Development Goals (SDGs) have identified gender-based violence as a key priority. This is both highlighted explicitly (SDG 5.2) and more broadly in terms of gender equality and empowerment of women and girls (SDG 5) (Garcia-Moreno & Amin 2016).

Although within the wider definition of gender-based violence, as a separate entity sexual violence according to the WHO (2021) is defined as any sexual act, attempt to obtain a sexual act, unwanted sexual comments or advances or acts to traffic, or otherwise directed, against a person’s sexuality using coercion, by any person regardless of their relationship to the victim, in any setting including at home and at work.

Gavey (2014) states that sexual violence is an umbrella term that refers to an inclusive category of sexual acts and experiences that are imposed, coerced or forced onto a person. Rape, attempted rape, sexual assault, sexual abuse, sexual violation and so on are all included.

Jansen (2016) refers to sexual violence as any sexual act or attempt to obtain a sexual act or unwanted sexual comments or acts to traffic that are directed against a person’s sexuality using coercion by anyone, regardless of their relationship to the victim, in any setting, including at home and at work. According to WHO (2021), rape that is part of sexual violence is described as physically forced or otherwise coerced penetration of the vulva or anus, using a penis, other body parts or an object attempted rape, unwanted sexual touching and other non-contact forms.

The impact of gender-based violence on physical and psychological wellbeing is wide ranging. This includes immediate physical injury as well as longer-term chronic ill health as a result of injury, acute and enduring psychological trauma, mental ill-health, substance and/or alcohol misuse, self-harm and suicide alongside secondary physiological health issues such as gynaecological, sexual health and gastrointestinal health problems (Feder et al. 2011).

The reasons for the high rates of sexual violence and assault in South Africa have been debated within the wider literature and includes gender inequality as well as the social and economic impact of apartheid. The threat of violence can have a deleterious effect on women’s citizenship, impacting the spaces in which women feel free to move, act and speak, feeding into institutional inequalities (Buiten & Naidoo 2016).

About 9516 cases of sexual assault were reported to the South African Police Service (SAPS) between April 2022 and June 2022; this is almost 500 fewer sexual assault cases reported compared to the same period in 2021 when the country was placed under Lockdown Levels 1 and 2 because of the outbreak of the coronavirus disease 2019 (COVID-19).

The recent crime statistics for sexual assault highlight that over 10 000 rape cases were opened with the SAPS between July 2022 and September 2022. From a sample of 8227 sexual assault incidents that were perused, it was determined that 5083 that is 62% of these incidents occurred at the residence of the victims or perpetrators, 1651 of the incidents occurred at public places such as streets, parks and beaches while 69 people were sexually assaulted at abandoned buildings (South African Government 2022a and 2022b).

About 73.8% of the total cases of sexual offences recorded by SAPS were rapes; this is an underestimation of the actual number as the underreporting of rape to the police is a well-known phenomenon (Abrahams & Gevers 2017).

While other sources suggested the statistics equate to a woman in South Africa being raped every 17 s (Connolly 2017). It has been further highlighted that up to half of all women in South Africa will experience a lifetime history of gender-based and/or sexual violence from a partner (Watt et al. 2017).

Bougard and Booyens (2015) affirmed that the subsequent treatment of women within the criminal justice system mitigates the under-reporting of sexual violence, which includes feelings of self-blame and fears among others.

It has also been identified that women in South Africa are also subject to so-called ‘rape stigma’, which may act as a further barrier to women formally reporting sexual violence and/or accessing health and support services (Jewkes et al. 2022). In their study, Holton, Joyner and Mash (2018) highlighted that in addition to the well-documented barriers to clinical care, for example, trust and confidentiality concerns and access to facilities including prohibitive costs of travel, a pivotal factor centred on an individual’s perception that their disclosure would be recognised and believed.

As a direct result of sexual violence, many women will require both immediate and ongoing specialist medical and psychological care and as such may present to a range of healthcare settings including emergency care departments (Sebaeng, Davhana-Maselesele & Manyedi 2016). In South Africa, the healthcare needs of women who have been subject to sexual violence are largely provided by designated public health facilities (Medecins Sans Frontieres 2017). However, 85% of designated facilities are hospital based, while 57% of facilities rely on doctors for conducting forensic examination, which could result in long waiting times for survivors (MSF 2017). While a number of these are specialised multi-agency facilities called Thuthuzela Care Centres (TCC) (Thuthuzela is an isiXhosa word meaning ‘comfort’) – TCC provides comprehensive medical and psychological care alongside legal and social assistance – a significant number of other facilities that support women who have experienced sexual violence offer mainly essential medical and clinical forensic care. The range and comprehensiveness of services available, however, may vary between different facilities (MSF 2017).

In addition, there is a limited availability of specialised forensic nursing training, and while the professional competencies of forensic nursing have been recognised by the South African Nursing Council (SANC), the Department of Health (DoH) does not acknowledge forensic nursing as a specialism. The non-recognition of forensic nursing training has potential implications in terms of remuneration and status for those who work in the forensic care environment (MSF 2017).

The role of health care professionals and those they work with is pivotal in providing physical and psychological support to victims of sexual violence in South Africa. Despite the nature of the work and the associated complexities, there is relatively little available evidence with regard to the experiences of those working in forensic care centres (FCCs) in South Africa. This study, which formed part of a larger doctoral study, aimed to address this knowledge and evidence gap.

### Aim

The overall aim of the study was to explore the experiences of healthcare staff working in FCCs who provide support to those who have experienced sexual violence in Tshwane, South Africa in terms of enablers and barriers and complexities of care provision.

## Research methods

Although recognised as diverse in nature, qualitative approaches to the research enquiry have been described as encompassing an interpretive and naturalistic approach to its subject matter and give priority to what the data contribute to important research questions or existing information (Avis 1995; Denzin & Lincoln 2002), underpinned by a philosophy that places the emphasis on the meanings that individuals attach to their social world (Bowling 2014). The study sought to provide an in-depth account of the experiences of healthcare staff working within the particular context of FCCs facilities in South Africa and a qualitative approach to the research enquiry was adopted. The research method was appropriate as it allowed the researcher to address the research questions, which were developed to be exploratory in nature.

As such, the researcher is by the very nature of this approach, a principal instrument of data collection, (Brewer 2000) interpretation and data analysis. Therefore, reflexivity is arguably pivotal in considering the role of the researcher in the actual production of the data (Davies 1999) and consequently the trustworthiness of the research endeavour (Morrow 2005). As nurses and researchers, the authors were mindful of the possible tensions inherent in conducting research in their own profession, the necessity to record experiences and reactions and to reflect on these as part of the research process.

### Research tool

A semi-structured interview guide was used to collect data, which focused on exploring the experiences of healthcare staff working in forensic care facilities providing support to those who have experienced sexual violence.

### Setting

The study was undertaken at two designated FCCs (A and B) of specific district hospitals situated in the Tshwane Metsweding District Region, which forms part of the six districts located in the Gauteng province in South Africa. The centres in this region were chosen as they serve two extensive geographical areas comprising of populations of around 200 000–300 000 people, respecctively (Gauteng Province 2016) and had high rates of reported cases of sexual violence (Government Gazette, Republic of South Africa [RSA] 2016).

Monthly statistics revealed that approximately 500 cases of violence are attended to between the two FCCs (Government Gazette, Republic of South Africa 2016). The Centre A typically received between 180 and 190 patients per month, while Centre B received an average of 290 patients per month. The highest number of cases seen were of sexual violence as compared to domestic violence cases. Healthcare staff who work in the FCCs are formally employed by the hospital and the primary health care centre, respectively. The FCCs offer the comprehensive services that are offered by the current Thuthuzela Centres.

### Study population and sampling strategy

The study population comprised all healthcare staff members working in both FCCs. A total of 12 participants agreed to take part in the study (Table 1), and this comprised healthcare staff from both FCCs. There were five members of healthcare staff in Focus Group 1 (FCC A) and seven members of healthcare staff in Focus Group 2 (FCC B). Participants in the study included nurses registered with SANC (*n* = 7), a registered medical practitioner (*n* = 1), counsellors (nurse auxiliaries) (*n* = 2) based in the FCCs and data capturers (*n* = 2) (responsible for initial reception and case history taking for individuals on arrival to the centres).

**TABLE 1:** Demographic characteristics of participants.

Criterion	Characteristic	Number†	%
Gender	Female	10	83.3
	Male	2	16.6
Professional category	Registered nurse	6	50.0
	Enrolled nurse	1	8.3
	Nurse auxiliary (counsellor)	2	16.6
	Doctor	1	8.3
	Data capturer	2	16.6
Number of years working at the site	5 months – 1 year	2	16.6
	2–5 years	4	33.3
	6–9 years	2	16.6
	10 and more years	4	33.3
Formal forensic training	Professional nurse	5	41.6

† , Participant number.

Purposive sampling was used to recruit and select the study participants (Brink, Van der Walt & Van Rensburg 2018). Initial contact was made by the researcher to the FCCs managers to discuss the study and its aim. Email correspondence along with study information was then circulated to all healthcare staff and a face-to-face follow-up visit was undertaken to explain the purpose of the study. All the 12 participants were included in the study as they were above 18 years and had experience of being directly involved with the victims of sexual violence. It is worth taking note that all healthcare staff met the inclusion criterion, and no one was excluded from the study.

### Data collection

Data were collected through two focus group interviews conducted in both FCCs. Furthermore, focus group interviews were chosen as they serve to elicit responses between the members of the group and enrich the conversation (Kumar 2011; Polit & Beck 2014) by allowing the expression of opinions and challenges encountered by healthcare staff while providing care and support to survivors of sexual violence.

With the participant’s permission, the focus groups interviews were audio-recorded and subsequently transcribed. All data were anonymised and stored in accordance with the research policy of the host university. Before the actual data collection commenced, a pre-piloted aide memoir was used by the researcher to test the efficacy of the tool among two healthcare staff. The findings from the pre-testing of the data collection instrument were used to modify some of the questions that were not clear, and the participants were included in the final study because of the limited number of healthcare staff in the centre.

Two focus group interviews were conducted at the FCCs at a time and date convenient to participants. The duration for each of the focus groups was between 2 h and 3 h. Data were collected between February and May in 2016 as part of a wider study. The researcher carried out both focus group interviews in English, which is the official language of communication in the workplace. Data were collected until no new information emerged confirming data saturation. Refreshments were provided for both focus groups after the interview sessions to appreciate their participation in the study.

### Data analysis

In the present study, the analysis was informed by the Analytic Hierarchy Model (AHM) (Ritchie & Lewis 2003), which is described as having essentially three stages: data management, descriptive accounts and developing explanatory accounts.

Following transcription, focus group interview transcripts were individually read repeatedly by the lead author to gain an understanding of the meanings and nuances of the texts.

The data were coded independently by the authors and developing themes were reviewed and discussed to confirm and ensure representation of the range of views expressed by participants across the transcripts.

### Trustworthiness

In the present study, the researchers were engaged in the process of reading and rereading the data, coding, developing initial themes, revisiting the data and revising until final themes were identified and agreed. The themes identified are supported by the data as illustrated in the inclusion of quotes and accompanying narrative. Data triangulation was ensured by interviewing an adequate number of participants at different FCCs, until data saturation was reached, which was the point where there was informational redundancy when no new information emerged and by using field notes, observations and audio recordings.

### Ethical considerations

Ethical approval to carry out the study was granted by the Research Ethics Committee of the Sefako Makgatho Health Sciences University. An ethical clearance certificate was issued: MREC/H/175/2014. Permission to conduct the study was sought by the researcher and granted by the relevant authorities and research sites. Because of the sensitivity of the topic, specialist support was available to participants during the study. Informed consent was sought prior to the focus group interviews taking place. Confidentiality of study participants was maintained through the secure storage of consent forms, audio equipment and anonymisation of data in keeping with the host university’s storage and research protocols. Data will be0 stored for 8 years.

## Results

Three themes emerged from the analysed data are presented in Table 1. The verbatim codes of participants are indented, and participants are written in brackets as P followed by the number, for example (P1 RN).

### Description of participants

The characteristics pertained to gender, category, years of experience and forensic training. Out of the participants, five had formal forensic training while the others received in-service education, which was provided in house by those who have been trained in forensic nursing. It is worth noting that the participants who received formal training as well as those who only received in-service education experienced the same challenges in dealing and providing care to victims of sexual violence. Three themes emerged from the analysed data and are presented in Table 2.

**TABLE 2:** Summary of three emerging themes.

Themes	Description
1.	We help them to do away with the idea of self-blame: Everyday work
2.	Barriers to the accessibility of care: seeking alternative traditional remedies ***(muti)*** from traditional healers and working in an unconducive environment
3.	Compassionately sick at times: Emotional impact of forensic care work

### Themes

#### Theme 1: We help them to do away with the idea of self-blame: Everyday work

In this theme, we highlight that at first glance the role of those working in the FCCs environment may be viewed as encapsulated within the physical roles or tasks as described. However, further exploration also uncovered that underpinning everyday work was the more complex role of providing psychological care and support. Furthermore, the supportive role was not simply confined to those providing clinical care. As such, everyday work held the meaning of what was both the expected but also more hidden aspects of boundary and role fluidity.

Those in the study described a range of activities that formed part of their role within the FCCs. At the onset of the focus group interviews, the work described appeared to largely centre on routine clinical procedures. This involves performing particular clinical tasks such as supporting medical examinations, collection of samples such as blood human chorionic gonadotrophin (BhCG) and evidence for use in possible criminal proceedings as highlighted by the participants:

‘I reassure and explain procedures on what is going to happen to the patient, evidence procedures performed by the doctor, perform Beta Human Chorionic Gonadotrophin [*β-hCG*] pregnancy test and prepare packs from South African Police Services [*SAPS*].’ (P1, registered nurse [RN])

‘… [*I*]nterview the client and take history, assess the client, monitor vital signs, take blood samples, and assist the doctor with the examination.’ (P2, RN)

While counsellors within the FCCs described how they provided counselling and advice with regard to medication and follow-on treatment for those who had experienced sexual violence:

‘I do counselling and educate patients about treatment … but I also attend to rape and human bite cases … I counsel the person [*following rape*]. In most cases clients do not know their [*HIV*] status. So, it is my duty to make them safe.’ (P11, counsellor)

However, as the focus group interviews progressed, it became clear that a significant part of staff clinical role, and not just the dedicated counsellors, also involved providing counselling and support to patients who had experienced physical and sexual assault and abuse. This involved both recent incidents and supporting those who attended as a result of historical abuse. Moreover, it was also clear that clinical staff in the FCCs also sought to provide, through their care, a place of refuge as well as clinical care. For example, the following participant explains the care provided as ‘they have hope’:

‘… [*A*]nd importantly avoiding self-blame. When they leave here, they have hope that whatever happened to me, I got assistance here. The confi-pack that we give also makes a difference as they bath afterwards and feel cleansed before leaving. This makes us see a great difference between when they arrived and when they leave.’ (P9, RN)

In addition to the role of the counsellors, it was also clear that nurses felt that they were able to communicate with the support those who attended the FCCs on a more personal level and as such as the following participant highlights, forming a bond beyond that of a nurse:

‘… [*W*]e form that bond, that bond that is special that makes her feel like she is not talking to a nurse. She can even tell you about other problems at home and that’s when we identify most of the things.’ (P10, RN)

The support and counselling role undertaken by staff also extended to administrative staff members. Administrative staff (data capturers) explained that they were often the first point of contact for women who attended the FCCs:

‘… [*I*]f the mood is down or eyes are filled with tears, you cannot tell the patient come, come let’s open a file … we know that the patient still needs to be comforted.’ (P6, Data Capturer)

All of the clinical staff in the study had undertaken some form of professional preparation for the forensic environment. However, the content and duration of the training were not universal. Continuing professional development was also noted to be patchy among study participants:

‘I am trained in Counselling and Advanced Counselling Courses and Forensic Nursing. I attended a 10-day training [*programme*] and did practical’s at X and Y Hospitals. Now and then we also attend workshops on Counselling.’ (P8, RN)

‘Training was just on how to handle and take care of trauma victims. We underwent training for 10 days. The training was for 2 days per week and for now we have in-service education in between for taking care of the victims who have been traumatized.’ (P1, RN)

‘In our society, people think that abuse is only when the husband shoots the wife. In our society people normalise the situation. That is why I tell her you that we cannot take a decision on behalf of the client. When a husbands slap the wife or punches her on the face that is actually abuse. But the same client will say “my husband loves me”.’ (P6, data capturer)

The above quote from a participant in the present study perhaps most clearly encapsulates the multi-faceted phenomenon of sexual violence within the particular context of South Africa – but geographically it is by no means unique as sexual violence remains a significant global problem. With the inception of specialist care facilities for women who have experienced sexual violence in South Africa, undoubtedly the services have developed over the last four decades or so.

Moreover, as previously highlighted, while the data capturers were often among the first to encounter patients in the FCCs, they had not received any formal preparation for this role. This is an important omission and one that we return to in a later theme.

#### Theme 2: Barriers to the accessibility of care: Seeking alternative traditional remedies *(muti) from* traditional healers and working in an unconducive environment

It was clear from the focus group discussions that those who worked within the FCCs considered the work to be complex and difficult. Survivors face a number of stigmas when coping with the trauma of sexual assault. The biggest and most common stigma lies in responsibility. The nature and scope of these complex spaces ranged from what survivors’ experience when coping with the trauma of sexual assault, with the biggest and most common stigma lying in responsibility that is put on the survivor. It is because of the victim-blaming society that survivors often fear reporting their assault to law enforcement.

Study participants, for example, identified a number of societal barriers in providing effective care to women who attended the FCCs. Participants spoke of the stigma surrounding sexual violence and the perceived lack of privacy (women might be identified because of the lack of private space) within the FCCs as a barrier to women accessing care and treatment. These barriers may prevent either initial contact with services or inhibit follow-up care. For example, during the focus group interviews, the lack of follow-up among women was a recurring theme and not uncommon. Study participants also spoke of the ‘self-blame’ that women who attended the FCCs felt and how this acted as a further barrier to care:

‘We make sure that we spent some time with them, making sure that they understand about the happenings and procedures to be done; and that they have knowledge about treatment and importantly avoiding self-blame.’ (P9, RN)

Cultural practices and ***muti*** [muti is traditional African medicines] are still honoured in some social groups as opposed to Western medicines. While religious practices were also identified as approaches used as opposed to FCCs for some women who had experienced sexual violence. One of the participants for example highlighted the following by way of a barrier to the provision of medical care:

‘We have religious and cultural challenges. Some of the clients’ drink water [*anointed water*] from the church as medication and do not want to take the PEP [*post exposure prophylaxis to prevent infection*]. They say the water has been prayed for and it will make them heal. Whilst some take “*muti*” from traditional healers as treatment instead of PEP … They do not attend counselling sessions, and do not honour appointments with the social worker or the psychologist.’ (P1, RN)

In terms of physical barriers, the lack of resources (including staff) and information was noted by many of those in the study as a deficit to care provision. During data collection, the researcher noted that in one of the centres only, one consultation room was in use to conduct examinations for victims. This was because of the fact that it was not a fully equipped and developed centre as explained earlier under the setting section. The waiting area was open, which did not separate women attending for the first time from women who were attending for follow-up appointments and as the following participants highlighted:

‘This Centre has got many big problems. It needs space, it needs a confidentiality area. Everybody to have his or her own space […] Now we pass here going for tea and making noise whilst the doctor is busy with a patient.’ (P3, RN)

The shortage of staff willing to work within the FCCs was also identified as a key deficit in care delivery as this resulted in long waiting periods for clients:

‘The other challenge is the shortage of staff, and the structure is not conducive for victims.’ (P1, RN)

‘Another thing that we lack, like they say [,] is shortage of staff. We need a person who will assess and make a follow up on cases at the clinic [,] hand in hand with the police. We need a person who can do follow ups with patients that is during the visits at the clinic. i.e., A case manager who will liaise between the courts, patients and the clinic.’ (P6, Data Capturer)

However, despite these challenges the study participants further stated that they could not cut their services short and that they understood the importance of prolonged engagement. This included ensuring that those who attended felt that time was not rushed. Participants emphasised that as their role is to support those who attended that they could not allocate a specific time limit to see a client. Their aim was to make sure that the victim understood the situation they are faced with and that they felt supported:

‘We continue with counselling until the victim is seen by the doctor and until the victim feels ready to can disclose … You cannot say that you will stay with this client for so long … It depends on an individual … We give water to drink, if that is not helping, we leave her for a while. After some time, we try to re-examine again and if we still fail, we then give her some more time …’ (P8, PN)

‘There is a guideline that we follow. But you can add to it some information that is missing. But still like my colleague said, the period will differ depending on the individual.’ (P9, PN)

#### Theme 3: Compassionately sick at times: Emotional impact of forensic care work

Unsurprisingly, participants in the study also spoke of the personal and emotional impact of working in the FCCs. These effects were described in a number of ways. For example, study participants spoke of how disclosure by patients about their abuse left staff feeling ‘burdened’ through confidentiality requirements:

‘We have to give the patients the platform to release the burden that they come carrying. They come here with stressful situations. And very true, when they leave, we are the ones who are left with the burden. We have to practice confidentiality.’ (P5, counsellor)

‘*re imetswe [we are burdened here] [we need somewhere]* at least to offload.’ (P2, RN)

It was also clear that at times the burden became too great for staff to bear. Those in the study, for example, spoke of feeling ‘broken’ by the weight and nature of disclosure:

‘After attending to three assault cases you are broken … Previously we used to work alone. Now it is better with counsellors being available you have someone to talk to and share the experience. I used to breakdown, Sister X knows, I used to call her and say I am going home. It is emotionally draining.’ (P8, RN)

Moreover, those in the study also spoke of how they felt ‘compassionately sick’ at times in that they felt overwhelmed and unable emotionally to absorb any further traumatic disclosures. It was also clear that for those in the study their everyday experiences also impacted significantly on their relationships with their own family members:

‘We are sort of compassionately sick at times. There is a time when you feel like staying at home. You feel that you have carried [*enough*] here. We are parents as well. Imagine when a minor comes who explains to you that she has been sexually assaulted for more than a year since she was 9 years or 10 years old. As a mother she is explaining all that to you, and you have a 10-year daughter too. That can also happen to your 10-year-old child.’ (P6, Data Capturer)

Furthermore, working with and supporting those who had experienced sexual assault and abuse also exerted a personal cost to study participants beyond their everyday practice. It was clear that the parameters between professional and personal aspects of participant’s lives were often blurred. For example, many of those in the study spoke of heightened fear of strangers when travelling and heightened vigilance for the safety of family members and especially children:

‘You end up being overprotective with your own children. You no longer trust anybody. You want your kids to behave in a certain way and you are always suspicious. My children are all girls, I cannot trust male visitors and I therefore keep on asking myself if they go to play or visit somewhere not around me will they be safe or not. When you are home, you end up locking the gates all the time. People think that you are insane, and this is because of the situation that you are exposed to at work.’ (P8, RN)

‘With me when I bath my daughter, I ask her a lot of questions like “*ga wa gobala*”? Meaning “are you not hurt”? … Myself, I use a taxi on a daily basis. I now check the passengers when I climb [*in*] and when I find two men passengers, I become afraid and no longer relaxed.’ (P11, counsellor)

Finally, study participants spoke of the perceptions of their role among colleagues outside of the FCCs and the lack of support they felt that they received as a result. Participants associated this lack of support with the lack of understanding of what the forensic role entailed. For example, participants spoke of how colleagues viewed the FCCs as a relaxing place with no challenges – hence calling it a ‘rest area’ as it was furnished and had a television set (all of which were to promote the comfort of those attending the FCCs). This final point clearly illustrates both the multiple challenges faced by those working in FCCs – in terms of the nature of the work, the resource implications and the perceived status of their work within the broader spectrum of healthcare delivery:

‘I think people must come and ask me what we are doing in here, I will show and explain to them … [*post exposure prophylaxis to prevent infection*].’ (P1, RN)

‘You are always tired and traumatised when you work in here. My colleagues in other departments say that I do not want to work, I am comfortable and seated in the sofas with the TV [*television*] … For our colleagues to see the fridge, TV and sofas they say that we are comfortable. We are not comfortable, “*re bereka boima ka mo*” [*meaning: our working conditions are difficult in this Forensic Care Centre*]. Our colleagues do not understand our work environment. Other staff members must come and rotate in here to have a picture of what it looks like in here …’ (P2, RN)

## Discussion of findings

The aim of this study was to explore the daily experiences of the healthcare staff who provide care and support to those who have experienced sexual violence within the particular context of the FCCs in Tshwane, South Africa. This study illuminated a number of the key challenges from the perspectives of those providing care. The findings of the present study also highlight potential deficits within current care provision for those accessing services. In the present study, the role of clinical staff encompassed a range of physical tasks, for example, history taking, preparing clients for examination, the collection of samples and other forensic evidence.

However, in addition to the physical aspects of care provision, it was also clear that all members of staff provided a significant amount of psychological care and support, often referred to as ‘counselling’ for those who attended the FCCs. The focus of counselling was often largely towards calming clients in preparation for the physical examination.

The responsibility for the provision of this level of psychological support also extended to those who were on the front line including the data capturers who were not employed in clinical roles and had not received any formal training.

During the present study, it was noted that the psychological support offered on the first visit, alongside other care and support, was difficult to sustain as clients often did not attend follow-up appointments to the FCCs. Staff identified a number of possible reasons why clients did not attend follow-up appointments. A key issue identified by participants in the study was the perceived stigma that surrounded sexual violence, which subsequently acted as a barrier to clients wishing to disclose. Disclosure requires a trusting relationship between the victim and the person to whom the disclosure is made. Victims often feel trapped in the abuse, ashamed and being terrified of other people learning about what happened to them. Sadly, the fear silences the victims, perpetuating non-disclosure while the violence continues.

The FCCs in this study were situated within close proximity to wider hospitals or health services, and this may have directly affected perceptions among clients relating to privacy and confidentiality. Stigma has been cited elsewhere in the literature as a barrier to accessing services and appropriate care following sexual violence (Holton et al. 2018) and is acknowledged and interwoven by feelings of guilt and self-blame by the victims instead of correctly allocating responsibility for violence to the aggressor (Karakurt, Smith & Whiting 2014).

In terms of stigma, staff in the present study also highlighted how colleagues within the wider hospital or health environment did not understand or value the work that they were undertaking. As such, those working in the FCCs felt that they were judged by colleagues as not working hard enough or that their work was not specialist in nature. A report published by Medecins Sans Frontieres (2017), which examined gaps in forensic care for survivors of sexual violence in South Africa, has also drawn attention to the lack of recognition generally for those who work in forensic care environments. Recognition or the low-status role attributed to those working in FCCs, alongside the wider societal stigma that surrounds sexual violence in South Africa, clearly has the potential to directly affect staff morale and ultimately client experiences of care and support as noted in this present study.

It was clear in this present study that wider societal factors also exerted an impact on engagement with treatment and care. For example, staff spoke of the use of traditional medicines *(* ***muti*** *)* among those who attended the clinic as often in preference to the recognised prescribed medications. This was perceived by staff as a barrier to adherence or use of PEP and has been noted as such elsewhere within similar sensitive health contexts (Azia, Mukumbang & Van Wyk 2016). However, while adherence to physical treatment is important, limited engagement and follow-up may also act as a barrier to the uptake of psychological support post-trauma.

Azia et al. (2016) further agreed that in sub-Saharan Africa, there is an increase in the use of traditional concoctions, holy water and prayers as a cure for HIV and/or AIDS, preventing adherence to the recognised and free antiretroviral therapies.

While the number of survivors supported at centres varied throughout the day, the availability of staff was variable and was cited as not managed well for their workload, impacting negatively on the perceived quality of care provided. This often resulted in survivors having to wait for many hours to receive medical attention. Of note, our participants spoke of accepting to work longer with survivors to ensure that they received the most appropriate care regardless of the time taken.

Azia et al. (2016) conducted a study that looked at barriers to adherence to antiretroviral treatment in a regional hospital in Vredenburg with 18 patients. Human resource constraints might result in congestion, long waiting periods and patient dissatisfaction leading healthcare users to reject or not attend clinics to receive appropriate care and medication (Azia et al. 2016). The same sentiment is shared by Randa (2019), who indicated that it is important to allow unlimited time for caring for each victim, so that they are encouraged to talk freely and open up about the disclosure; however, this may prolong the waiting times for other victims. In their study, Johnson, Mahlalela and Mills (2017) also identified that the biggest gap in the services and satisfaction at the FCCs were the long waiting periods and associated experience of physical discomfort (hunger, feeling dirty, exhaustion and pain) and insufficient ongoing counselling. In order to avoid this in FCCs, healthcare staff must provide quality care that is associated with holistic management of victims of sexual assault by taking into consideration their physical, social and emotional aspects of care (Ravhura 2014). Therefore, regular information and updates should be provided to survivors while waiting to be attended to by staff at the centres.

The mental healthcare especially post-sexual trauma care was felt to be important by staff but largely limited in its use to the initial visit to the FCCs. More widely within the literature, a number of commentators have drawn attention to the paucity of psychological care and mental health support services for victims of sexual violence in South Africa. Abrahams and Gevers (2017) reported that services often prioritise clinical or forensic management and care. Limited availability of responsive mental health care has been identified as impacting negatively both immediate and longer-term psychological well-being (Vetten 2015). Sexual assault can be psychologically devastating for survivors while it has also been underlined that attention to the mental health needs of sexual assault victims remains poor (Abrahams et al. 2012). In their study, Tarzia et al. (2017) also found that poor mental health in women attending Australian general practices was associated with sexual violence, even when a broader definition was used. Women most commonly reported experiencing public harassment or flashing, unwanted groping and being coerced into having sex.

Finally, it was also clear that the work undertaken in the FCCs exerted a substantial personal cost for those in the study. Study participants spoke of the ways in which their experiences had impacted on their views and trust of others outside of the work environment. These findings resonate with Goldblatt (2009) who also described the struggle to separate the professional from the private in her research involving nurses working with abused women.

Healthcare practitioners including nurses display their emotions at work while interacting with patients. This emotional labour or emotion work is an intrinsic part of their work role in which they display the appropriate emotions, as they sometimes hide or fake felt emotions, although this might be difficult in some situations (Badolamenti et al. 2018).

As mentioned by Abrahams and Gevers (2017), it is a difficult task when offering support to survivors of sexual violence because of inadequate resources to provide mental health support for them, as well as the struggle of the healthcare workers themselves while dealing with work stress and trauma. It was also evidenced in this study that the preparation and training of staff working with sexually assaulted survivors was beneficial towards the optimal delivery of sexual violence services.

### Implications

The findings of the research have the potential to impact on national resource investment, education and training and ultimately the way in which care is experienced by women who access services. While recognised as a global concern, sexual violence has been identified as a large-scale problem in South Africa. In South Africa, the healthcare needs of women who have been subjected to sexual violence are largely provided by designated public health facilities. The main findings highlight the multiple challenges of providing care and support and this is framed within the wider societal context of violence against women.

### Strengths and limitations of the study

It is acknowledged that this was a small-scale study undertaken in two FCCs in one province in South Africa. However, the present study also illuminated a number of key issues for those working with victims of sexual violence in an area, which has received little research attention to date and within the particular context of South Africa. As such, the findings from the present study offer a contribution to the limited available evidence base and have resonance for similar settings alongside the wider debates around future care and service development in this field.

## Conclusion

This study highlighted that there is a gap for greater emphasis on sustained care pathways, especially mental health and psychological support for survivors post-trauma. It will require substantial investment and resources, both financial and human resources including debriefing sessions to minimise the effects of indirect trauma for survivors or those working in these FCCs. However, alongside any front-line service or care delivery developments the wider context of policy reform and systemic societal change with regard to prevailing assumptions and attitudes towards gender-based violence need to be considered and are pivotal to this evolving health agenda in South Africa.

### Recommendations

#### Policy makers

Policy makers can use the findings to appreciate the challenges that healthcare staff working at the forensic facilities encounter and re-consider the existing policies that may contribute to them being supported by implementing interventions for their support to avoid vicarious trauma.

#### Future research

Further research needs to be conducted to address the challenges encountered by healthcare staff while providing care to victims of sexual violence, so as to provide long-term solutions to the identified challenges.
