# Corrigendum: Experiences of healthcare staff in forensic care facilities supporting sexual violence survivors, in Tshwane, South Africa

**DOI:** 10.4102/curationis.v47i1.2591

**Published:** 2024-02-13

**Authors:** Moreoagae B. Randa, Julie McGarry

**Affiliations:** 1 Department of Public Health, Faculty of Health Care Sciences, Sefako Makgatho Health Sciences University, Pretoria, South Africa; 2 Health Sciences School, University of Sheffield and Sheffield Teaching Hospitals NHS Foundation Trust, Sheffield, United Kingdom

In the published article, Randa, M.B. & McGarry, J., 2023, ‘Experiences of healthcare staff in forensic care facilities supporting sexual violence survivors, in Tshwane, South Africa’, *Curationis* 46(1), a2374. https://doi.org/10.4102/curationis.v46i1.2374, the authors neglected to include the funder South African Medical Research Council, SIR:058, to Randa MB.

The authors apologise for this error. The correction does not change the study’s findings of significance or overall interpretation of the study’s results or the scientific conclusions of the article in any way.

